# Pulse-Gauging

**Published:** 1895-06

**Authors:** 


*Pulse-Gauging*. By GEORGE OLIVER, M.D. Pp. xiv., 174. London: H. K. LEWIS. 1895.

Dr. Oliver's interesting and valuable observations upon the calibre of arteries may possibly stimulate investigation in a new direction in the scientific study of heart affections. By means of his arteriometer, Dr. Oliver shows how the calibre of the radial artery varies in health with the different postures of the body, during exercise and after meals. He also shows in various pathological conditions that it fails to act as in health. For example, the artery is normally larger in the standing position than in the recumbent, but in many forms of disease the reverse is the case.

Dr. Schott at Nauheim, as is well known, has introduced a system of treating heart-disease by baths and exercises. He has attributed the beneficial effect of his methods to reflex stimulus of the heart. That may be in part true; but the experiments of Dr. Oliver with a pulse-pressure gauge, another instrument which he has devised, seem to indicate that various stimuli act much more readily upon the blood-vessels than upon the heart itself. The action of the heart of course depends largely upon the condition of the peripheral circulation, and it may be that in many instances in order to treat the heart it is necessary to treat the blood-vessels. All practitioners, even those of the smallest experience, must know how disappointing rest with digitalis may be, and it is to be hoped that methods such as those of Dr. Schott, carefully applied in the light of knowledge gained by observations such as those of Dr. Oliver, may open a new era in the treatment of cardiac disease.

